# Exploring the Impact of Altitude on Bacterial Communities in Informally Produced Artisanal Colonial Cheeses: Insights from 16S rRNA Gene Sequencing

**DOI:** 10.3390/microorganisms13051116

**Published:** 2025-05-13

**Authors:** Wemerson de Castro Oliveira, Anderson Santos de Freitas, Jeferson Aloísio Ströher, Neila Silvia Pereira dos Santos Richards, Maria Beatriz Prior Pinto Oliveira, Magnolia Martins Erhardt

**Affiliations:** 1 REQUIMTE/LAQV, Department of Teaching, Research, and Extension, Federal Institute of Education, Science and Technology, Lajeado 95910-016, RS, Brazil; 2 Department of Food Science and Technology, Federal University of Santa Maria, Santa Maria 97105-900, RS, Brazil; neila.richards@ufsm.br (N.S.P.d.S.R.); magveterinaria@gmail.com (M.M.E.); 3 Center for Nuclear Energy in Agriculture, University of São Paulo, Piracicaba 13416-000, SP, Brazil; andersonfreitas@usp.br; 4 Institute of Food Science and Technology, Federal University of Rio Grande do Sul, Porto Alegre 90010-150, SP, Brazil; jeferson.stroher@hotmail.com; 5 REQUIMTE/LAQV, Department of Chemical Sciences, School of Pharmacy, University of Porto, 4050-313 Porto, Portugal; 6 Department of Food Science and Technology, State University of Rio Grande do Sul, Encantado 95960-000, RS, Brazil

**Keywords:** environmental factors, food microbiology, metabarcoding, NGS

## Abstract

Artisanal cheese microbiota plays a key role in defining its sensory properties, safety, and overall quality. Environmental factors, including altitude, influence microbial communities by altering temperature, pressure, and radiation levels, yet the extent of these effects on cheese microbiota remains unclear. Here, we investigated bacterial composition, diversity, and correlation patterns in artisanal cheeses produced along an 800 m altitudinal gradient in Southern Brazil using 16S rRNA gene sequencing and bioinformatic analyses. Diversity analysis showed no significant variation in microbial community structure across altitudes, suggesting that overall bacterial diversity is stable within this range. Physicochemical parameters, including moisture, pH, and fat content, also exhibited no statistical differences. However, differential abundance analysis revealed that while broad taxonomic profiles remained consistent, small differences were observed at deeper taxonomic levels, with *Lactiplantibacillus*, *Psychrobacter celer*, and *Lactococcus raffinolactis* showing altitude-associated variations. These findings suggest that altitude exerts only a subtle influence on cheese microbiota, primarily at lower taxonomic ranks. This study contributes to understanding environmental effects on cheese microbiomes, with potential applications for optimizing production and quality control in diverse altitudinal regions.

## 1. Introduction

Brazil produces approximately 1 million tons of cheese per year, with small producers expanding to account for around 20% of total production, predominantly artisanal [1]. Brazilian artisan cheeses reflect the country’s cultural and geographical diversity, with more than 30 recognized varieties. Among the most representative are the cheeses Araxá, Canastra, and Serro (Southeast), Caipira (Midwest), Marajó (North), Manteiga and Requeijão (Northeast), and Sul Serrano and Colonial (South), which are sources of lactic acid bacteria (LAB) used in the development of starter cultures [2,3,4,5]. Colonial cheese emerged in Rio Grande do Sul, in family production units, as part of the process of Italian and German immigration that took place in the second half of the 19th century. Initially intended for family consumption, surplus milk was processed into cheese, a practice predominantly carried out by women, who not only looked after the livestock, but also produced the cheese by hand, using raw milk, rennet, and salt [6].

Currently, artisanal colonial cheese, largely produced with raw milk, still faces regulatory challenges in Brazil. Current legislation requires that the product be matured for a minimum of 60 days, or that its microbiological safety be proven through laboratory tests before it can be sold [7,8]. For cheeses matured for less than this period, pasteurization of the milk is mandatory, compromising the product’s microbiological and sensory complexity [5]. Although many small producers, especially family farmers, still use raw milk, the lack of standardization of production processes makes it difficult to formalize the marketing of cheese [9,10]. Corroborating this, Lai et al. [11] state that standardizing the production of artisanal cheeses, such as Pecorino Romano, prevents the growth and survival of pathogenic bacteria, ensuring greater microbiological safety.

Traditionally, artisanal colonial cheese is produced with milk from the Jersey or Holstein breeds, undergoing curd heating (35–40 °C) and maturing for between 30 and 75 days, resulting in a cheese with scattered eyes and a yellowish color [2]. In some cases, the product is marketed after a shorter maturation of 7 to 10 days [12], considered to occur under high-humidity conditions [7]. High-moisture, unpasteurized cheeses are more susceptible to contamination, which favors the survival and proliferation of pathogenic microorganisms [13].

It is therefore essential to define a standardized protocol to evaluate the effectiveness of cheese ripening, taking into account its specific characteristics [14]. Normative Instruction no. 002 [15] establishes specific criteria for the identity and quality of colonial cheese, such as compact texture, smooth rind, fat content between 45% and 59.9%, minimum maturation of 10 days (≥5 °C), and sanitary control of raw milk. However, informality still prevails in the sector, which results in a non-standardized product with significant variations in its physical, chemical, and microbiological attributes. This reality highlights the need for public policies that balance food safety, the appreciation of artisanal cheeses, and the strengthening of family farming, with a particular focus on knowledge about the product’s microbiota, to guarantee greater standardization and quality control of this product, which is so significant for the region.

Microbial communities profoundly influence artisanal cheese production, which plays a key role in defining the final product’s sensory properties, safety, and overall quality. Its microbiome consists of a complex network of bacteria that contribute to fermentation, flavor development, and textural characteristics. It is shaped by environmental factors such as milk source, production practices, and ripening conditions [16,17,18,19,20]. These environmental factors not only determine the microbial composition but also directly impact the adaptability and interactions within the microbiota, reflecting the complexity of the microbial ecosystem and the evolution of cheese characteristics. Understanding these interactions between species, and not just their presence and abundance, is essential for ensuring the quality and safety of the final product [21]. Furthermore, as highlighted by Neviani et al. [21], microbial diversity and minority strains play crucial roles in the stability and functionality of the microbial consortium, allowing the preservation of the sensory and technological properties of artisan cheeses throughout the fermentation process. Therefore, in-depth exploration of these interactions and the appropriate choice of starter cultures become fundamental to optimizing performance and guaranteeing the quality of the final product, while maintaining the desirable characteristics of the natural cultures [22,23,24,25].

Among the environmental factors that shape the microbiota of artisanal cheeses, altitude has emerged as a key variable due to its impact on local climate conditions and available food sources for cows. Altitude influences temperature, atmospheric pressure, and ultraviolet (UV) radiation, all of which can affect bacterial growth and community structure. In cheesemaking, these environmental variations may lead to differences in microbial succession during fermentation and ripening, potentially altering the biochemical processes that define the final cheese characteristics [26,27,28]. However, studies investigating the impact of altitude on artisanal cheese microbiota remain limited, particularly in regions with moderate altitude variations.

Microbial adaptation to altitude involves a range of physiological and genetic responses that allow bacteria to thrive under different environmental conditions. At higher altitudes, where temperatures tend to be lower, psychrotolerant bacteria may become more dominant, while oxidative stress due to increased UV radiation could favor species with protective mechanisms against cellular damage. In contrast, lower-altitude environments, with milder conditions, may support a different microbial composition dominated by mesophilic species. These shifts in microbial communities may have direct consequences on the sensory profile and safety of artisanal cheeses produced at different elevations [29].

This study aims to evaluate how altitude influences bacterial composition, diversity, and microbial correlations in artisanal cheeses produced within an 800-m elevation range in Southern Brazil. Using 16S rRNA gene sequencing, we hypothesize that cheeses produced at higher altitudes will exhibit greater microbial diversity and an increased presence of psychrotolerant bacteria, while cheeses from lower altitudes will be characterized by the dominance of mesophilic species. The findings of this study will contribute to a better understanding of the role of altitude in shaping artisanal cheese microbiota, with potential applications in optimizing cheesemaking practices and improving quality control across different production regions.

## 2. Materials and Methods

### 2.1. Characterization of the Region and Collection of Cheese Samples

The study was carried out in the Vale do Taquari region, located in the central region of the state of Rio Grande do Sul, Brazil. This region is characterized by a strong influence of European colonization, with municipalities of Germanic origin standing out.

Thirteen samples of fresh homemade cheeses, characterized by high moisture content and produced from raw milk, were collected. The samples were obtained directly from small commercial establishments and street markets located in different municipalities in the Vale do Taquari region. The products analyzed come from small local producers who sell their cheeses informally, without the presence of health inspection, food safety seals, or any type of official quality control. The cheeses were subjected to a maturation period ranging from seven to ten days before analysis.

For analysis purposes, the samples were categorized into three groups according to the altitude of the production region—low altitude (<100 m), medium altitude (100–450 m), and high altitude (>450 m)—by Erhardt et al. [12]. Table 1 describes information about the cheeses and their classification according to the altitude of the municipality where the sample was produced.

After collection, the samples (Figure S1) were packed in cool boxes and kept refrigerated during transportation to the Food Analysis Laboratory at the State University of Rio Grande do Sul (UERGS) to carry out pH analyses using a Datalogger-Digital Instruments pH meter (Phmeter model P1000, Phox, Colombo, Brazil), moisture (drying at 105 °C) and fat percentage (Gerber butyrometer) measurements, and metagenomics. For the metagenomic analysis, 200 g aliquots of each cheese were sent under refrigerated conditions to a specialized and accredited laboratory in Florianopolis, Brazil (Neoprospecta^®^), where they were processed and subjected to sequencing of the genetic material.

### 2.2. DNA Extraction and Sequencing

DNA extraction and sequencing were performed according to the proprietary protocol developed by the outsourced laboratory (Neoprospecta Microbiome Technologies, Florianopolis, Brazil), as described by Erhardt et al. [12]. High-throughput sequencing was performed by amplifying the V3–V4 regions of the 16S rRNA gene using the following primers: 341F with the sequence (CCTACGGGRSGCAGCAG) and 806R with the sequence (GGACTACHVGGGTWTCTAAT) [30,31]. The libraries were subsequently sequenced using the MiSeq Sequencing System (Illumina Inc., San Diego, CA, USA). The sequences obtained were discovered through the Sentinel pipeline. Initially, fastq files were assessed for Phred quality (PQ) using FastQC software (v.0.11.8). Then, fastq files were subjected to a process of removing primers and low-quality sequences (Phred < 20) using proprietary software developed in Python (v.3.6). All raw reads were deposited in Sequencing Read Archive (SRA) under the project number PRJNA1238316.

### 2.3. Read Processing and Statistical Analyses

All bioinformatic and statistical analyses were carried out in the R environment (version 4.3.0) using the RStudio software (version 2023.09.1+494) [32]. The code for the analyses carried out in this study can be found publicly on GitHub (https://github.com/FreitasAndy/CheeseAndAltitude, accessed on 10 march 2025). The figures were produced using the ggplot2 and ggpubr packages [33], and some of these figures were edited (i.e., changing colors and fonts) using the Inkscape 1.3.2 program. As the data did not fit the normal distribution, we proceeded with an analysis suitable for non-parametric data. We used the Kruskal–Wallis test followed by Dunn’s post hoc test [34] to test for differences unrelated to the microbiota.

The raw reads from sequencing were analyzed using the DADA2 pipeline [35], considering acceptable sequences with an average quality score higher than or equal to 30. The filtered reads were grouped into amplicon sequence variants (ASVs) and associated with taxonomy using the SILVA v. 138.1 database [36]. The resulting ASV table was imported into both a phyloseq [37] object and an R6 object from the microeco package [38] for subsequent analysis.

Alpha diversity was calculated considering the number of different taxa identified in each sample (observed diversity), weighted diversity (Shannon index), and the dominance of taxa (inverse Simpson’s index), considering a 95% confidence level using the Kruskal–Wallis test followed by Dunn’s post hoc test. Correlations between altitude and diversity index were calculated using Kendall’s coefficient (*p* < 0.05) aiming to not force the linear correlation [39]. Beta diversity was calculated by transforming the dataset into a centralized logarithmic ratio (CLR), to reflect the compositional structure of the data [40]. The data were ordered using Euclidean scaling, and the non-metric multidimensional scaling was plotted on the first two axes. Significance was calculated by permutational multivariate analysis of variance (PERMANOVA), at a significance level of 5% and 999 permutations, using the vegan package [41]. Differential abundance was carried out using ALDEx2 [42]. We considered as differentially abundant all taxa with effect size higher than 1 and a *p*-value calculated by the Wilcox test lower than 0.1.

## 3. Results and Discussion

### 3.1. Eight Hundred Meters Are Not Enough to Change the Microbial Diversity in Artisanal Colonial Cheeses

**Figure 1:**
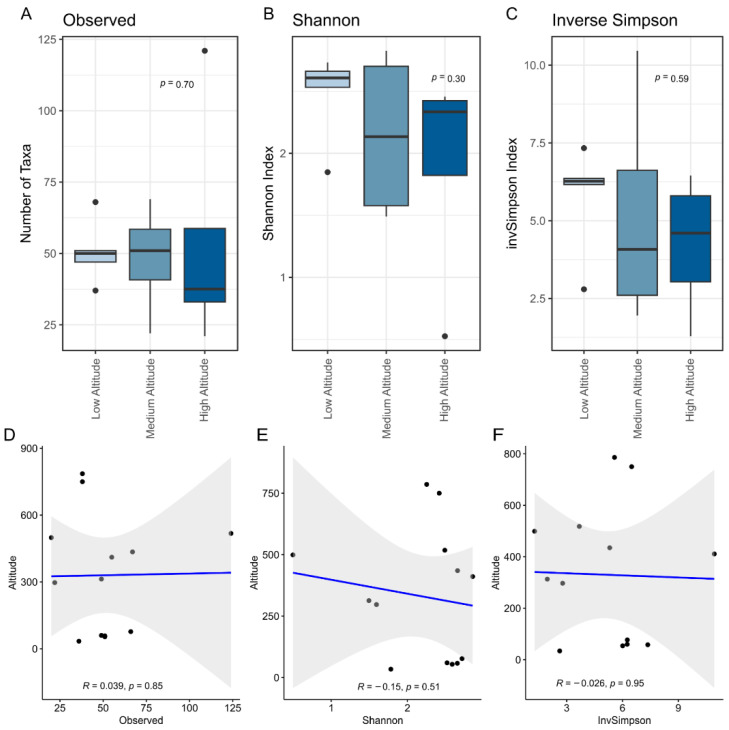
Alpha diversity metrics of microbial communities in artisanal colonial cheeses across different altitudes. (**A**) Observed number of taxa, (**B**) Shannon diversity index, and (**C**) inverse Simpson index are compared among cheeses produced at low, medium, and high altitudes. Boxes span the first to third quartiles; the horizontal line inside the boxes represents the median. Whiskers extending vertically from the boxes indicate variability outside the upper and lower quartiles, and the single black circles indicate outliers. Statistical comparisons were carried out using the Kruskal–Wallis test. (*p* > 0.05) across altitudes. (**D**–**F**) Correlation analyses between altitude and each diversity metric. Each dot represents a sample. The blue line indicates the fitted model and the grey area represents the model error. R and *p*-value were calculated using the Kendal correlation coefficient.

Microbial diversity in artisanal colonial cheeses is not significantly affected by an altitudinal gradient of up to 800 m. Alpha diversity metrics, including observed taxa richness (Figure 1A), Shannon diversity index (Figure 1B), and inverse Simpson index (Figure 1C), show no significant differences between cheeses produced at low, medium, and high altitudes. Correlation analyses between altitude and microbial diversity indices (Figure 1D–F) also showed weak and non-significant relationships (*p* > 0.05), suggesting that the microbial community structure in these cheeses remains stable despite differences in elevation. These findings align with previous studies indicating that cheese microbiota is primarily shaped by milk composition, fermentation practices, and aging conditions rather than by environmental factors such as altitude [43], although Erhardt et al. found a variation in the abundance of LAB on artisanal colonial cheese according to the elevation level, determining a specific fingerprint [12]. The species *Enterococcus italicus* and *Lactococcus lactis* were more abundant at high altitudes, also presenting seven species exclusive to this elevation, and *L. raffinolactis* were present in greater specificities at low altitudes, with eight specific species. The resilience of microbial communities in traditional cheeses has been attributed to the dominance of well-adapted core taxa, such as LAB, which can buffer environmental fluctuations [44], as well as differences in vegetation composition (the food for dairy cows) that become more pronounced with altitude, extending over thousands of meters [45].

### 3.2. Bacterial Taxa Are Resilient to Altitudinal Variation in Artisanal Colonial Cheeses

**Figure 2:**
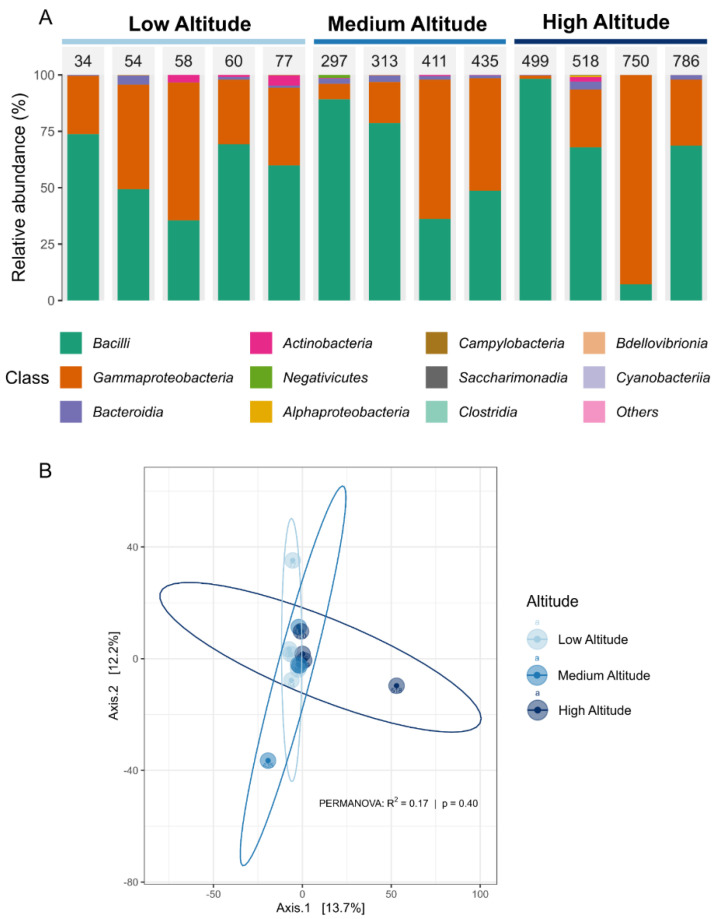
Microbial distribution and beta diversity. (**A**) Frequencies of the ten most abundant classes in each sample by altitude. (**B**) Overall comparisons of samples based on principal coordinate analysis (PCoA) measured by the Euclidean distance. Points closer to each other represent similar microbial communities, while points farther from each other represent dissimilar microbial communities. The statistical significance for the differences among altitudes was calculated by per MANOVA and is shown in the figure.

The taxonomic composition of the core microbiota revealed that *Bacilli* and *Gammaproteobacteria* were the dominant classes across all sampled altitudes. Still, their distribution was not consistent along the dataset, with the dominance of one or another not following a pattern (Figure 2A). Despite the observed variability in the relative abundance of microbial taxa, beta diversity analyses indicated no significant differentiation among the three altitude groups (low, medium, and high) (Figure 2B). These results reinforced the notion that an 800 m variation in altitude is insufficient to drive substantial shifts in microbial diversity. Previous studies have shown that environmental factors such as temperature, humidity, and feeding practices can influence microbial communities in artisanal cheeses [24,46,47]. However, the lack of significant changes in any diversity analysis suggests that other determinants, such as raw milk microbiota, cheese-making practices, and maturation conditions, may play a more decisive role in shaping the microbial structure. These findings highlight the resilience of the cheese microbiota to moderate altitudinal variations and underscore the importance of local production practices in defining microbial fingerprints [48,49].

**Figure 3:**
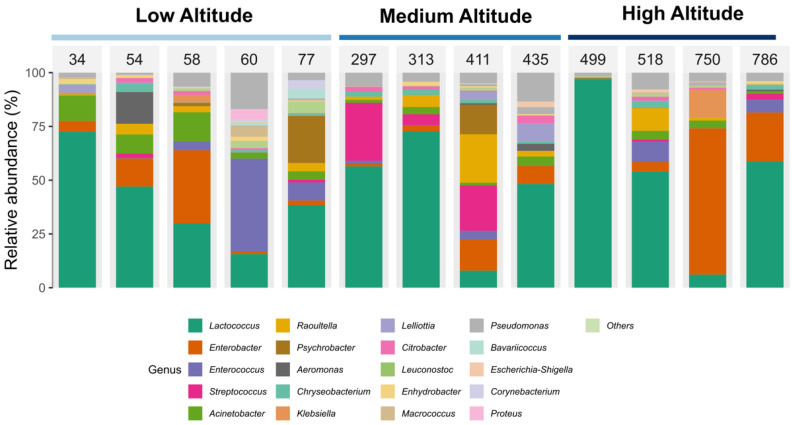
Distribution of genera in cheese samples produced at different altitudes.

The analysis of the taxonomic distribution at the genus level (Figure 3) in the samples from different altitudes revealed high microbial diversity, without identifying a consistent pattern associated with altitudinal variation. The genus Lactococcus was the most abundantly represented in most samples, regardless of altitude, followed by occasional increases in Enterococcus. A higher prevalence of the genera Streptococcus was also observed at medium altitudes and of Enterobacter at high altitudes. The absence of a homogeneous microbial profile among the samples suggests that the cheeses analyzed were not subjected to pasteurization and that there was no addition of industrial starter cultures composed of standardized strains during the fermentation process.

### 3.3. Physicochemical Variables Are Stable in Artisanal Colonial Cheeses Across Altitudes

**Figure 4:**
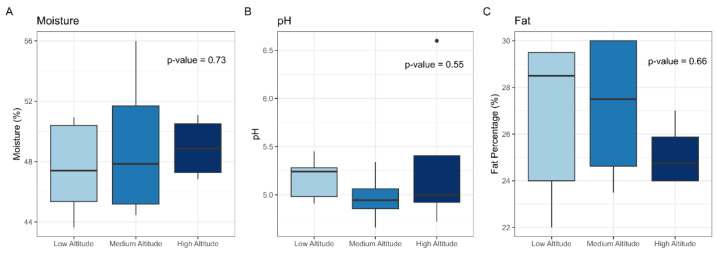
Physicochemical properties of artisanal colonial cheeses at different altitudes. Boxplots represent (**A**) moisture content (%), (**B**) pH, and (**C**) fat percentage (%) across low, medium, and high altitudes. The *p*-values were calculated using the Kruskal–Wallis test (*p* < 0.05).

The analysis of physicochemical parameters (moisture, pH, and fat percentage) also revealed no significant differences among cheeses produced at low, medium, and high altitudes (Figure 4). These characteristics remained stable despite an 800 m altitudinal gradient, also suggesting that local environmental factors other than altitude, such as milk composition and cheesemaking practices, may play a more prominent role in defining these physicochemical traits. Previous studies have shown that variations in moisture and fat content in artisanal cheeses are often more closely linked to the specific microbial activity during ripening rather than external abiotic factors [50]. Additionally, the stability of pH across altitudes supports the notion that dominant microbial groups, particularly *Bacilli* and *Gammaproteobacteria*, may contribute to maintaining a consistent acidification process, reinforcing the microbial resilience observed in the beta diversity results, once the studied cheese came from close regions of the Southern Brazil.

Studies on artisanal colonial cheese highlight variations in its physicochemical attributes. Erhardt et al. [51], when evaluating artisanal colonial cheese aged for 7, 15, and 60 days in the Taquari Valley, Brazil, found that cheeses aged for 7 and 15 days had an average moisture content of between 46.0% and 54.9%, classifying them as high-moisture cheeses. After 60 days, the moisture was reduced to 23.38%, categorizing them as low-moisture or hard cheeses. The pH ranged from 4.67 to 6.16 (7 days), 4.52 to 5.46 (15 days) and 4.71 to 5.18 (60 days). Ströher et al. [48] reported a median moisture content of 41.70 ± 1.51 g/100 g, with values ranging from 39.38 g/100 g (January) to 46.06 g/100 g (July). Benincá et al. [52] observed moisture ranging from 44.22% to 51.10%, classifying the cheeses as medium- and high-moisture, according to Brazilian legislation [7,8]. These variations can be attributed to the lack of standardization in the artisanal process, salting, pressing pressure, and ripening [53].

### 3.4. Small Differences Regarding Altitude Can Be Found Only at Deep Taxonomic Levels

Despite the lack of differences in the previous analyses, differential abundance analysis revealed that some specific bacterial taxa exhibited significant variations between cheeses produced at low and high altitudes, with these differences being detected primarily at deep taxonomic levels. At the phylum level, Actinobacteriota showed a significantly higher relative abundance in cheeses from low altitudes compared to high-altitude samples, but no significant differences were observed at the class, order, or family levels, suggesting that altitude does not broadly impact the overall microbial composition of artisanal cheese (Table 1).

At the genus level, *Lactiplantibacillus* showed a higher abundance in cheeses from high altitudes. Similarly, at the species level, *Psychrobacter celer* and *Lactococcus raffinolactis* exhibited a notable increase in low-altitude samples compared to high-altitude ones. *L. raffinolactis* was previously associated with low-altitude cheeses [12] and is commonly associated with fermented food and probiotics, as well as most of the *Lactococcus* species [54]. *P. celer* is a species first isolated from seawater, with the ability to survive in saline conditions, such as inside a cheese [55]. Finally, *Lactiplantibacillus* is a non-pathogenic genus common in the gut microbiota of animals [56]. The differential abundance of specific taxa highlights potential niche adaptations that warrant further investigation, particularly regarding functional roles in cheese maturation and flavor development. One example is the artisanal Serrano cheese (SAC), produced with raw milk in the Campos de Cima da Serra-RS region, at approximately 1400 m above sea level, where, after production, the bacterial genera *Lactococcus* and *Enterococcus* predominate [24]. Despite the lack of differences in the previous analyses, differential abundance analysis revealed that some specific bacterial taxa exhibited significant variations between cheeses produced at low and high altitudes, with these differences being detected primarily at deep taxonomic levels. At the phylum level, *Actinobacteriota* showed a significantly higher relative abundance in cheeses from low altitudes compared to high-altitude samples, but no significant differences were observed at the class, order, or family levels, suggesting that altitude does not broadly impact the overall microbial composition of artisanal cheese (Table 2).

Several studies have identified and characterized the microbiota of artisan cheeses around the world, revealing a great diversity of microorganisms involved in the fermentation and ripening processes. This diversity is highly variable, reflecting a number of factors, such as the production and ripening conditions, which have already been described. Interactions between LAB and other microorganisms, such as yeasts and fungi, play a crucial role in shaping the sensory and functional characteristics of the final product. Regional and cultural variations in the microbiota highlight the complexity and uniqueness of artisan cheeses, making them a relevant field of study for understanding the impact of this microbial diversity on product quality [57,58,59,60].

Dairies and small producers of artisanal cheeses have invested in enhancing their production chains, and innovation in traditional foods is a strategic factor for regional and global economic growth [61]. In this context, Azevedo et al. [62] investigated the marination of colonial cheese in wine and vinegar, showing that this process influences maturation and alters its quality attributes compared to the control sample (traditional colonial artisanal cheese). Therefore, understanding the microbiota of colonial artisanal cheese under different production conditions is essential for the region studied.

André et al. [63] analyzed colonial cheeses from supermarkets in Francisco Beltrão-PR,t identifying that LAB isolated from them have probiotic properties, including tolerance to acids and bile salts, and antimicrobial activity against pathogens. These characteristics are promising for small producers in the region and indicate potential for application as probiotics, although additional in vitro and in vivo studies are needed to validate their efficacy. Myazaki et al. [64], through metataxonomic analysis, revealed the presence of microorganisms naturally found in milk and different varieties of cheese. Among the species identified, *L. casei*, *L. helveticus*, *Bifidobacterium psychraerophilum*, *Enterococcus durans*, *L. plantarum*, and *L. lactis* stand out for their probiotic potential and application as natural antibacterial agents in fermented dairy products. In addition, next-generation probiotics were detected, including species from the genus *Enterococcus* spp. These findings reinforce the relevance of metabarcoding as an essential tool for preliminary screening of the microbiota and for targeting the isolation of species of interest.

## 4. Conclusions

This study provided insights into the influence of altitude on the microbiota of artisanal cheeses produced within an 800 m altitudinal range in Southern Brazil. While overall bacterial diversity and community structure remained stable across different altitudes, subtle differences were observed at deeper taxonomic levels, with specific bacterial taxa exhibiting differential abundance. We rejected, in part, our hypothesis that environmental variations associated with altitude have a major impact on cheese microbial composition, likely due to the resilience of core bacterial communities and the standardization of traditional production practices. Furthermore, physicochemical parameters such as moisture, pH, and fat content did not significantly vary with altitude, reinforcing the notion that microbial and compositional stability may be maintained within this range. This study contributed to the knowledge of environmental factors shaping cheese microbiomes and highlighted the potential for refining production strategies to enhance product quality and consistency across diverse geographical settings.

## Figures and Tables

**Table 1:** Information about artisanal colonial cheeses produced informally in the southern region of Brazil.

Sample	Altitude (m)	Classification	Raw Milk	Cheese
LJ	34	Low	Yes	Colonial type
AM	54	Low	Yes	Colonial type
EN	58	Low	Yes	Colonial type
MU	77	Low	Yes	Colonial type
RC	60	Low	Yes	Colonial type
RV	297	Medium	Yes	Colonial type
NB	313	Medium	Yes	Colonial type
AG	411	Medium	Yes	Colonial type
PT	435	Medium	Yes	Colonial type
DR	499	High	Yes	Colonial type
VC	518	High	Yes	Colonial type
AV	750	High	Yes	Colonial type
IL	786	High	Yes	Colonial type

**Table 2:** List of microbial taxa with significant difference between high and low altitude group of cheese samples from Southern Brazil.

Taxonomic Level	Taxa	Rab ^a^ Low Altitude	Rab ^a^ High Altitude	Overlap ^b^	Effect ^c^	*p*-Value ^d^
Phylum	*Actinobacteriota*	1.80	−2.75	0.11	1.01	0.04
Class	-	-	-	-	-	-
Order	-	-	-	-	-	-
Family	-	-	-	-	-	-
Genus	*Lactiplantibacillus*	−3.56	3.98	0.07	−1.59	0.06
Species	*Psychrobacter celer*	4.47	−4.59	0.10	1.14	0.06
	*Lactococcus raffinolactis*	8.81	−4.36	0.14	1.09	0.06

^a^ Relative abundance normalized by centered log-ratios. ^b^ Confusion in assigning an observation to a high- or low-altitude group. ^c^ Effect size of the difference, median of difference between groups on a log base 2 scale/largest median variation within groups; positive values indicate a higher abundance in the low-altitude group, whereas negative values indicate higher abundance in the high-altitude group. ^d^ The expected value of the Wilcox test *p*-value. Table includes all amplicon sequence variants (ASVs) with effect  >1 and *p*-value  <  0.1.

## Data Availability

All raw reads were deposited in Sequencing Read Archive (SRA) under the project number PRJNA1238316.The code for the analyses carried out in this study can be found publicly on GitHub (https://github.com/FreitasAndy/CheeseAndAltitude).

## Supplementary Materials

The following supporting information can be downloaded at: https://www.mdpi.com/article/10.3390/microorganisms13051116/s1. Figure S1. Artisanal Colonial cheese sold informally in southern Brazil.
