# Supplementary material for: Exploring the Impact of Altitude on Bacterial Communities in Informally Produced Artisanal Colonial Cheeses: Insights from 16S rRNA Gene Sequencing
Source: Microorganisms. 2025 May 13;13(5):1116. doi: 10.3390/microorganisms13051116 (PMC12114159; doi:10.3390/microorganisms13051116)
Supplement: Supplementary file 1 [file microorganisms-13-01116-s001.zip › microorganisms-3572463-supplementary.pdf]

microorganisms

Article

Exploring the Impact of Altitude on Bacterial Communities in Informally Produced Artisanal Colonial Cheeses: Insights from 16S rRNA Gene Sequencing

Wemerson de Castro Oliveira [1,2,*,†], Anderson Santos de Freitas [3,†], Jeferson Aloísio Ströher [4], Neila Silvia Pereira dos Santos Richards [2], Maria Beatriz Prior Pinto Oliveira [5,*] and Magnolia Martins Erhardt [2,6]

Supplementary Figure S1

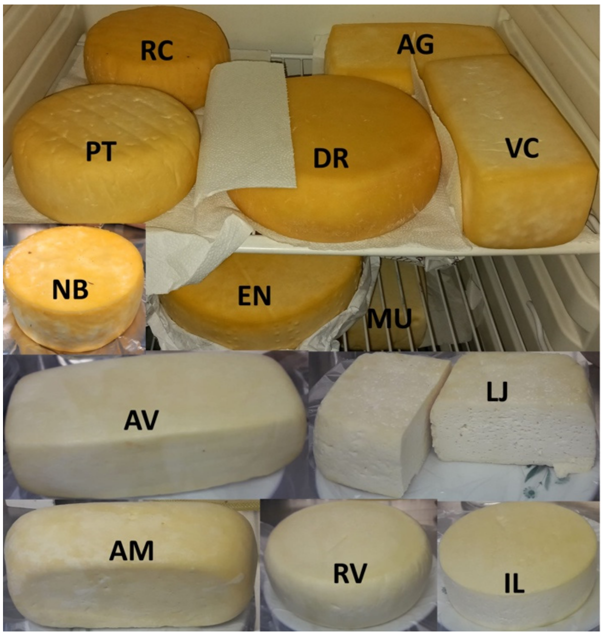

Supplementary Figure S1. Artisanal Colonial cheese sold informally in southern Brazil.
